# Oscillatory “temporal sampling” and developmental dyslexia: toward an over-arching theoretical framework

**DOI:** 10.3389/fnhum.2014.00904

**Published:** 2014-11-07

**Authors:** Usha Goswami, Alan J. Power, Marie Lallier, Andrea Facoetti

**Affiliations:** ^1^Department of Psychology, Centre for Neuroscience in Education, University of CambridgeCambridge, UK; ^2^Basque Centre on Cognition, Brain and LanguageSan Sebastian, Spain; ^3^General Psychology, University of PaduaPadua, Italy

**Keywords:** dyslexia, oscillations, reading, phonology, attention

Human cognitive systems such as language represent the sensory world as unitary. For example, the “speech signal” is perceived as a single auditory stimulus, and different visual features and textures in the visual field are perceived holistically as unitary objects. Yet sensory neuroscience demonstrates the diversity of encoding in the neural systems supporting sensory perception. Different aspects of sensory information are processed in parallel, often at different timescales and by different populations of neurons. Further, research into the function of *neuronal oscillations* (e.g., Buzsáki and Draguhn, 2004) reveals a key role in processing sensory information. In this special issue, we investigate the developmental implications of one neuroscientific theory based on oscillations that is relevant to reading education and developmental dyslexia, the “temporal sampling” framework (hereafter TSF, Goswami, 2011). The TSF identifies specific oscillatory mechanisms that may be impaired in dyslexia. We expect that deeper understanding of neural mechanisms of information-processing will eventually enable deeper understanding of developmental disorders of learning, such as dyslexia. Developmental dyslexia is a disorder in the acquisition of successful reading and spelling skills that is found in ~7% of children across cultures.

The ability to read and write—the achievement of literacy—is one of the most complex and sophisticated cognitive skills developed by the brain. Literacy skills develop as a result of direct teaching, usually in childhood, and become fluent during years of practice. By adulthood, most brains have read millions of words. This makes it difficult to disentangle cause from effect when studying sensory and neural factors. Dyslexia is usually only diagnosed after 2–3 years of schooling, when the brain has already had considerable experience of reading and reading tuition. Dyslexia is diagnosed when children who are receiving adequate teaching and who have no overt sensory or neurological problems fail to develop fast, efficient and age-appropriate reading and spelling.

The TSF was proposed as the neural basis for the extraction of phonological information from speech via auditory oscillatory encoding (Goswami, in press). Based on work by Poeppel, Greenberg, Giraud, Ghitza and many others (Giraud and Poeppel, 2012, for a recent summary), the TSF linked “sampling” of the speech stream by auditory cortical networks operating at different timescales or oscillatory frequencies (delta, theta, gamma) to the emergence of phonological (linguistic) coding of speech by children. Poeppel and others argued that cortical oscillations enabled the representation of different temporal rates of amplitude modulation in the complex speech signal. These temporal frequency bands yield complementary “windows” of information relating to cognitive linguistic units such as syllables (theta rate) and phonemes (gamma rate, see Poeppel, 2003). Accordingly, the TSF proposed that atypical oscillatory sampling at one or more temporal rates in children with dyslexia could cause phonological difficulties in specifying linguistic units such as syllables.

Phonological difficulties in dyslexia are related to difficulties in the accurate perception of amplitude “rise time” (related to modulation rate). The TSF proposed that atypical oscillatory entrainment at *syllable-relevant* rates of amplitude modulation (delta [~ stressed syllable rate], theta [~ syllable rate]) could be one neural cause of the “phonological deficit” found in children and adults with dyslexia across languages and orthographies. This theory is about early developmental mechanisms, nevertheless impaired oscillatory sampling in auditory cortex could, over developmental time, lead to atypical functioning of the left-lateralized “reading network” identified in many fMRI studies of older children (Richlan, 2012, for a recent overview; Clark et al., 2014, for a relevant longitudinal study). This should be true across languages. Indeed, rise time perception is impaired in English, French, Spanish, Hungarian, Finnish, Chinese, and Dutch dyslexic children (see Goswami and Leong, 2013, for an overview). The “phonological deficit” in dyslexia is found in all of these languages, and manifests as difficulties in oral tasks such as recognizing prosodic stress, counting syllables, and counting or deleting *phonemes* (the smallest phonological units in a language; Ziegler and Goswami, 2005, for a cross-language review). These and other phonological tasks are considered by some of the auditory-based contributions to the special issue (Lehongre et al., 2013; Power et al., 2013; White-Schwoch and Kraus, 2013; Sela, 2014 this issue).

The aim of this special issue, however, was to simultaneously invite colleagues who work on visual sensory processing to consider whether atypical oscillatory “temporal sampling” may explain the pervasive visual processing deficits in dyslexia reported in many orthographies (e.g., Facoetti et al., 2010; Lallier and Valdois, 2012). Visual and auditory sensory theories of dyslexia are typically considered to compete with each other, indeed a recent review counted 12 competing theories of developmental dyslexia (Ramus and Ahissar, 2012). The act of reading of course depends upon many visual processes. Examples are (for alphabetic orthographies) serial letter recognition, visual grouping of repeating letter patterns in familiar words, and the left-to-right (or in some orthographies, right-to-left) horizontal linear tracking of print. Practice in reading (reading experience) will obviously train the brain in aspects of visual processing related to reading. Such visual practice is necessarily reduced in children with dyslexia (reading is effortful, so the child reads less). Disentangling the effects of reading experience on the brain across the many different sensory and cognitive components that support the development of reading and writing is thus experimentally challenging. Nevertheless, by studying particular aspects of non-linguistic visual processing in isolation (such as magnocellular function, or eye movements), research can begin to disentangle cause from effect in developmental dyslexia.

In this special issue, a number of the different aspects of impaired visual and visuo-spatial attentional processing found in dyslexia are studied and possible relations with oscillatory temporal sampling are considered (see De Luca et al., 2013; Lallier et al., 2013; Conlon et al., 2013; Gori et al., 2014; Ruffino et al., 2014; Varvara et al., 2014 this issue). Theoretically, these contributions consider whether spatiotemporal sampling of information by the visual system may be impaired in dyslexia (see Pammer, 2014; Vidyasagar, 2013 this issue). Indeed, Vidyasagar argues that a visual sampling impairment may be *primary* to the auditory difficulties in dyslexia documented by other contributors, a provocative claim which requires longitudinal studies. In fact, in order to establish the possible causal role of different visual and auditory sensory processes to reading development, and to identify their sequential contributions during the developmental learning trajectory, a range of developmental research designs are required.

At minimum, evidence is required that:

1. the sensory/neural deficit precedes being taught to read
2. the sensory/neural deficit affects aspects of cognitive development other than reading (e.g., musical development for auditory deficits, conceptual development for visual deficits) in predictable ways
3. the sensory/neural deficit can be demonstrated when children with dyslexia are compared to younger children whose *reading skills* are matched with the dyslexics (this research design aims to equate the effect of reading experience on the brain; the *reading level match* research design)
4. developmental trajectories are followed in longitudinal studies, exploring the complex interplay of auditory and visual sensory/neural and cognitive processes during the development of reading, thereby establishing the developmental primacy of the candidate deficit
5. the sensory/neural deficit is consistent across different languages and orthographies
6. training the candidate deficit has demonstrable effects upon subsequent reading development

Longitudinal studies, beginning before reading is taught and carried out across languages, are enormously important to the field (e.g., Boets et al., 2011; Franceschini et al., 2012). Sensory/neural deficits may change over developmental time. Perhaps a sensory factor critical for early development becomes less relevant when studying older children, or is no longer apparent when studying adults. Sensory/neural deficits may also *manifest* differently in different languages, for example as a consequence of factors such as orthographic grain size (e.g., the phonemic grain size is practiced by readers of alphabetic languages, whereas Japanese readers practice the syllable grain size) or phonology (e.g., rhythmic or phonetic differences, such as whether a language has many sonorant phonemes and is syllable-timed, such as Spanish, or many plosive phonemes and is stress-timed, such as English). Reading difficulties may be comorbid with other difficulties such as attention-deficit-hyperactivity disorder (ADHD); the possible effects of co-morbid disorders on sensory processing must be taken into account (Thaler et al., 2009). The studies in this special issue document and calibrate some of these aspects of auditory and visual processing that seem to be important in developmental dyslexia. Incorporating all of these aspects of sensory processing into oscillatory studies will be the next task for developmental research into dyslexia.

## Conflict of interest statement

The authors declare that the research was conducted in the absence of any commercial or financial relationships that could be construed as a potential conflict of interest.
